# LncRNA FAM30A Predicts Adverse Prognosis and Regulates Cellular Processes in Colorectal Cancer via Modulating miR-21-3p

**DOI:** 10.5152/tjg.2024.23465

**Published:** 2024-07-01

**Authors:** Guoxiang Ye, Yanxiang Chen

**Affiliations:** Department of Oncology, Friendliness Hospital Yangzhou, Yangzhou, China

**Keywords:** Colorectal cancer, FAM30A, miR-21-3p, prognosis, metastasis

## Abstract

**Background/Aims:**

Colorectal cancer (CRC) is a prevalent gastrointestinal cancer with high incidence and mortality rate. lncRNAs could regulate the expression of miRNAs and further affect cancer development. Previous research has suggested that FAM30A is involved in various cancer. We aimed to investigate the function of FAM30A in the prognosis of CRC and its underlying molecular mechanisms.

**Materials and Methods:**

Matched tissues were collected from 107 CRC patients. FAM30A was measured by quantitative real-time transcription polymerase chain reaction and its clinical significance was evaluated by its correlation with patients’ prognosis and clinicopathological features. Furthermore, the dual luciferase reporter assays were employed to assess the interactions between FAM30A and miR-21-3p and to evaluate the role of miR-21-3p in regulating the tumor suppressor effects of FAM30A. Cell proliferation and metastasis were evaluated by the Transwell assay and cell counting kit-8 assay.

**Results:**

FAM30A level was markedly decreased in CRC tissues (*P* < .001). A prominent association was observed in FAM30A with tumor–node–metastasis stage (*P* = .022), carcinoembryonic antigen (*P* = .027), and differentiation (*P* = .043) of CRC patients. The lower the FAM30A level was associated with the lower the survival rate of the CRC patients (log rank *P* = .034). FAM30A could negatively modulate miR-21-3p (*P* < .001), and the overexpression of FAM30A significantly suppressed CRC cell proliferation and metastasis (*P* < .001). The suppressive function of FAM30A overexpression was mediated by miR-21-3p.

**Conclusion:**

FAM30A can be considered a poor prognostic indicator in CRC. Decreased FAM30A can promote the proliferation and metastasis of CRC cells by negatively regulating miR-21-3p.

**Main Points**

- Downregulated FAM30A in colorectal cancer predicts the deteriorating disease development and adverse prognosis.
- FAM30A serves as a tumor suppressor in colorectal cancer, inhibiting tumor cell growth and metastasis.
- miR-21-3p was negatively regulated by FAM30A and mediated the functions of FAM30A in regulating colorectal cancer cells.

## Introduction

Colorectal cancer (CRC) is the most common gastrointestinal cancer with the third most incidence and second most fatal cancer in the world.^1^ It has been proposed that the accumulation of mutations in proto-oncogenes and oncogenes in colonic stem cells was the main contributor to the emergence and progression of CRC.^2,3^ Besides, other risk factors also increase the risk of CRC, like age, diet, and lifestyle.^4,5^ In some developed countries, the majority of CRC patients are early diagnosed and the early endoscopic resection showed a significant effect on the recurrence of CRC. Yet nearly half of all new CRC cases are diagnosed at advanced stages.^6^ The main treatments for CRC include local excision, radiotherapy, and chemotherapy.^7^ Lymph node infiltration and distant metastases have occurred as the disease progresses to an advanced state. Thus the overall survival rate of CRC patients is modest. Among patients diagnosed with metastatic CRC, the 5-year survival rate is less than 20%.^8^ Distant metastasis and the cellular stemness feature of the cancer cells are the main causes of the worse prognosis for colorectal.^9^ Hence it is urgent to explore new independent prognostic factors and find new therapeutic targets for CRC.

Long non-coding RNA (lncRNA) with a length of over 200 nucleotides is unable to code proteins and performs multiple biological functions by regulating gene expression at the transcriptional, translational, and post-translational levels. Recent findings demonstrated that lncRNAs participate in the pathogenesis of various tumors through signaling pathways, including cell proliferation, migration, and invasion.^10^ Furthermore, many lncRNAs could act as miRNA sponges to impact their expression and further regulate cancer development.^11^ With the development of bioinformatics, there have been an increasing number of studies devoted to identifying differentially expressed lncRNAs in CRC, which were considered candidates for biomarkers. Based on the GSE196006, GSE104178, and GSE104836 datasets from the GEO database, a total of 12 dysregulated ncRNAs in CRC were filtered. Except for ncRNAs without supporting evidence, CRNDE, CADM3-AS1, CCAT1, and FAM30A were finally focused, where FAM30A was a novel member that has not been reported in CRC. The function of FAM30A has been disclosed in various cancers, including gastric cancer, laryngeal squamous cell carcinoma, and lung adenocarcinoma.^12-14^ In addition, FAM30A also could affect the proliferation and migration of acute myeloid leukemia and laryngeal squamous cell carcinoma cells.^13,15^ FAM30A was also demonstrated to be relevant to immunomodulatory processes.^16-18^ Thererfore, FAM30A was hypothysized as a novel potential biomarker for CRC, which was confirmed in the present study.

microRNAs (miRNAs) are also members of ncRNAs with small endogenous single strands, which assume a highly critical role in CRC. A growing amount of evidence revealed that lncRNAs were the critical component of competing endogenous (ce) RNA networks that could regulate protein coding and translation via sponging micro (mi) RNA in cancer.^19,20^ For example, miR-21-3p was suggested to be upregulated in CRC and showed facilitated effects on cell movement through epithelial–mesenchymal transition.^21,22^ Moreover, increasing expression of miR-21-3p has been suggested to correlate with the early T-stages of CRC.^23^ Intriguingly, miR-21-3p was also predicted as a direct ceRNA of FAM30A from the lncRNASNP v3 database (http://gong_lab.hzau.edu.cn/lncRNASNP3#!/). Whether miR-21-3p was involved in the regulatory effect of FAM30A on CRC progression was also assessed.

## Materials and Methods

### Clinical Samples

Tissue specimens, including tumor tissues and normal paracancerous tissues, were gathered from 107 CRC patients who were treated at Friendliness Hospital Yangzhou. Significantly, the patients had no other major illnesses except CRC, and no patient had been treated with anticancer therapy prior to diagnosis. The research was approved by the Ethics Committee of Friendliness Hospital Yangzhou (approval number: 2016110, date: November 2016). Written informed consent was also secured from the patients. The postoperative survival of the patients was acquired over 5 years via a follow-up survey on the telephone or through outpatient review. The gathered data was utilized in Cox regression analyses and Kaplan–Meier analyses.

### Cell Culture

Cell lines CCC-HIE2, SW837, SW1463, LoVo, and colo205 were obtained from the Chinese Academy of Sciences Cell Bank (Shanghai, China). All cells were cultured in the recommended media containing 10% fetal bovine serum (FBS) at 37℃ in 5% CO_2_.

### Cell Transfection

LoVo and SW837 cell lines were transfected with plasmids comprising FAM30A, miR-21-3p mimic, and miR-21-3p inhibitor. Transfection was performed at room temperature using Lipofectamine 2000 (Invitrogen, Carlsbad, CA, USA). The transfection efficiency was assessed by detecting the level of FAM30A and miR-21-3p after 48 hours of transfection.

### Luciferase Reporter Assay

The luciferase reporter assay was performed to evaluate whether FAM30A could sponge miR-21-3p. The interplay between FAM30A and miR-21-3p was assessed by subcloning the wild-type (WT) or mutant (MUT) 3’-untranslated region (3′-UTR) of FAM30A into the pmiRGLO vector (cat. no. E1330; Promega Corporation). Subsequently, LoVo and SW837 cells were co-transfected with miR-21-3p mimic or an inhibitor. Following 48 hours of transfection, luciferase activity of FAM30A was measured with a dual-luciferase assay kit (Promega, Madison, WI, USA).

### Quantitative Real-Time Polymerase Chain Reaction

The total RNA was withdrawn using TRIzol (Invitrogen). Quantitative real-time transcription polymerase chain reaction (qRT-PCR) was performed on StepOne Plus (Applied Biosystems, Foster City, CA, USA) with iTaq Universal SYBR Green Supermix (Bio-Rad, Hercules, CA, USA) to assess FAM30A and miR-21-3p. The references for FAM30A and miR-21-3p were GAPDH and U6, respectively, and the levels of them were calculated using the 2^−ΔΔct^ method.

### Cell Counting Kit-8 Assay

Cells were grown for 0, 24, 48, and 72 hours in 96-well plates with a complete medium. 10 μL of cell counting Kit-8 AssayCCK8 was then incorporated into each well. Then 10 μL of CCK8 was spiked into each well. Two hours after the addition of CCK8, the optical density values were read at 450 nm.

### Transwell Assay

Cells were vaccinated into the upper chamber of a 24-well Transwell plate (precoated with Matrigel for invasion assay) and supplied with FBS-free DMEM. Simultaneously, medium containing 10% FBS was placed in the bottom chamber. After 24 hours of incubation, cells on the subsurface of the upper chamber were fixed and stained. Migrating and invading cells from 5 randomly selected fields were counted with a microscope.

### Statistical Analysis

All data was organized using the Statistical Package for the Social Sciences version 23.0 (IBM Corp., Armonk, NY, USA) and GraphPad Prism 9.0. All measurements were from at least 3 replications, and data were expressed as mean ± standard deviation. The correlation between FAM30A and the clinicopathological characteristics of the patients was performed using the *χ* ^2^ test. *P* < .05 indicated statistical significance.

## Results

### The Level of FAM30A in Colorectal Cancer Tissues

**Figure 1.**
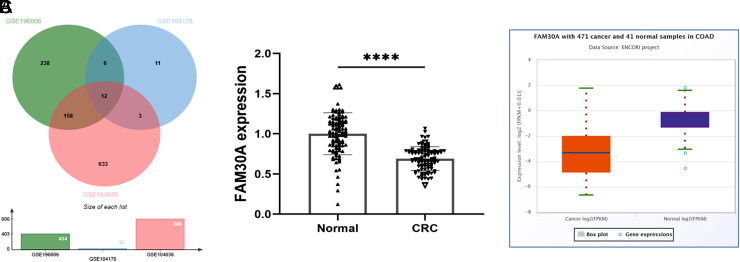
Dysregulated ncRNAs in colorectal cancer (CRC) were enriched from GSE196006, GSE104178, and GSE104836 datasets from the GEO database (A). FAM30A level in collected CRC tissues was lower than normal tissues (B) consistent with data from the starBase database (C). ^****^ *P* < .0001

According to GSE196006, GSE104178, and GSE104836 datasets from the GEO database, a total of 12 abnormally expressed ncRNAs were enriched (Figure 1A). In the tissues gathered from the patients, the level of FAM30A was remarkably lower than normal paracancerous tissues (Figure 1B), which is in concordance with the results queried in the starBase database (https://rnasysu.com/encori/panGeneDiffExp.php, Figure 1C).

### FAM30A in Relation to Clinicopathologic Features and Clinical Prognosis of Patients

Depending on the average level of FAM30A in CRC tissues, patients were divided into 2 cohorts. The high-FAM30A cohort was composed of 52 individuals, comprising 34 males and 18 females. Meanwhile, the low-FAM30A cohort included 55 individuals, comprising 34 males and 21 females. Statistically significant correlations were discovered between FAM30A and TNM stage (*P* = .022), CEA (*P* = .027), and differentiation (*P* = .043) in CRC patients (Table 1).

**Figure 2.**
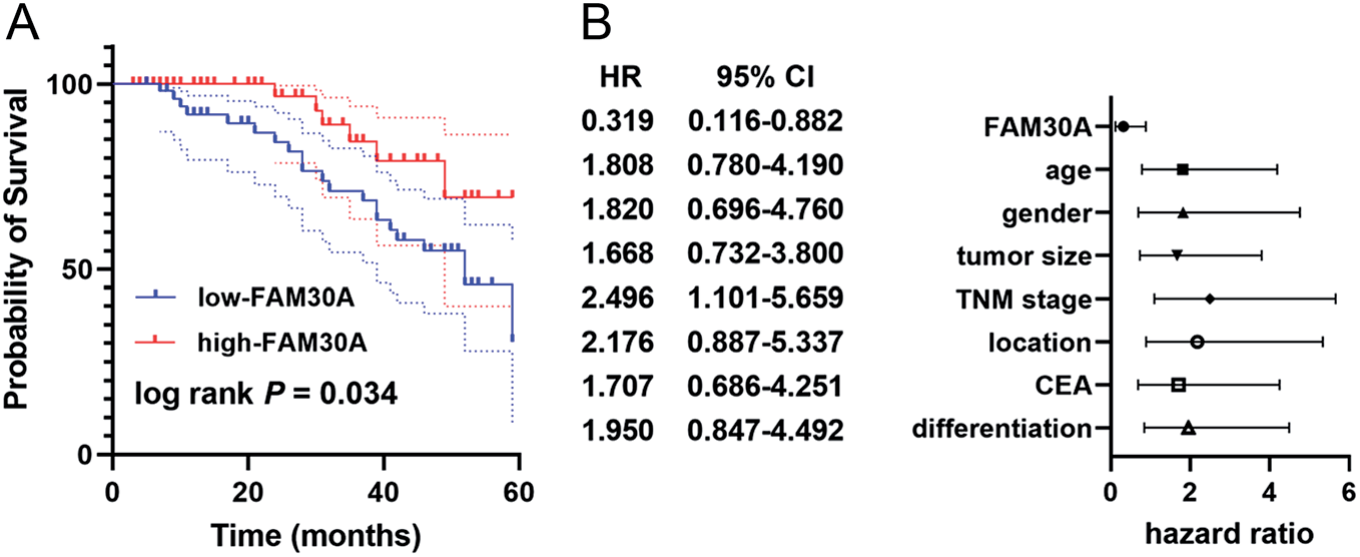
Kaplan–Meier followed by (A) the log-rank test assessing the overall survival rate of patients in 60 monthsCox regression analysis assessing the prognostic value of clinicopathological features and FAM30A (B).

The lower the FAM30A level showed a significant association with the lower the survivability of sufferers (log rank *P* = .034, Figure 2A). Concurrently, FAM30A was an independent prognostic factor (hazard ratio (HR) = 0.319, 95% CI = 0.116-0.882) along with TNM stage (HR = 2.496, 95% CI = 1.101-0.659) and location (HR = 2.176, 95% CI = 0.887-5.337, Figure 2B).

### Modulation of miR-21-3p by FAM30A

**Figure 3.**
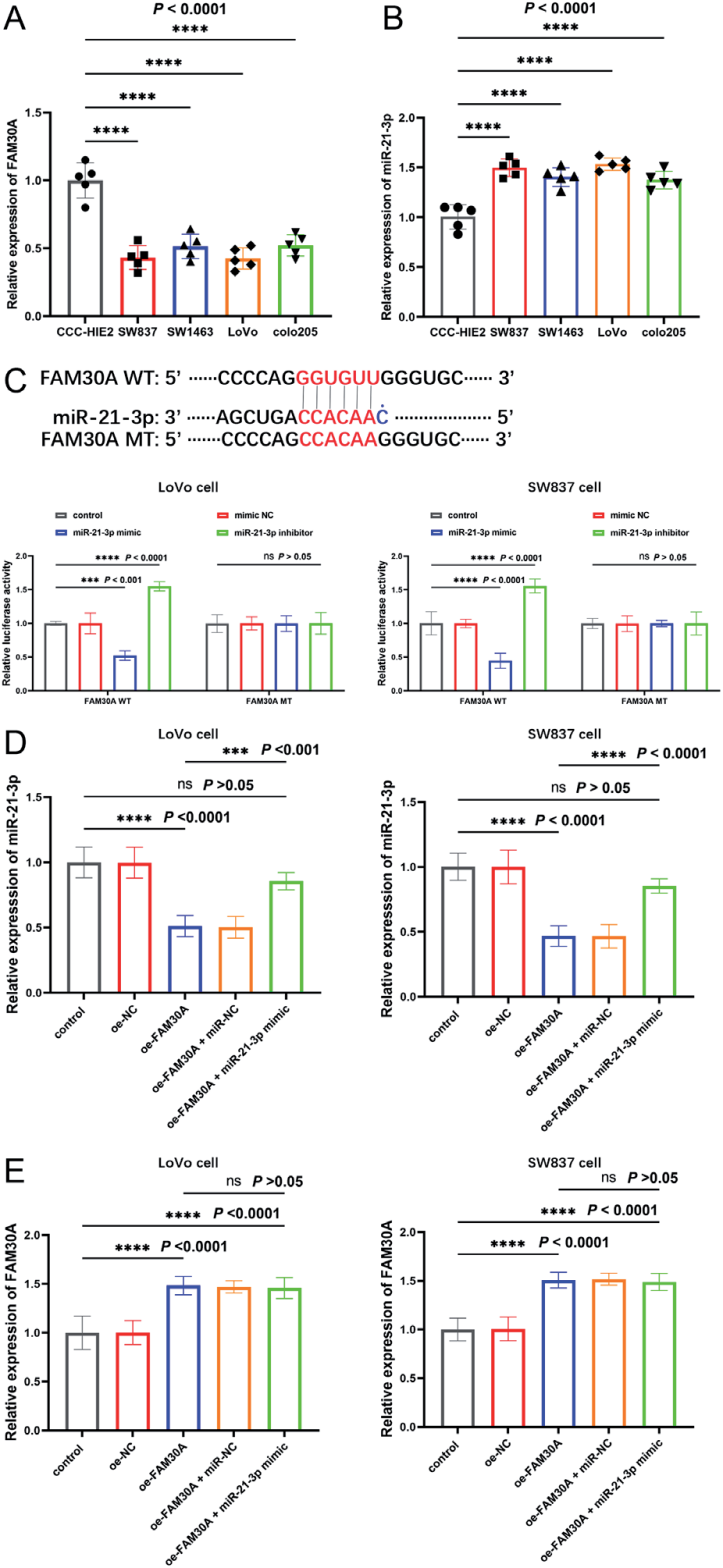
(A) Expression of FAM30A and (B) miR-21-3p in colorectal cancer (CRC) cell lines in comparison to CCC-HIE2. FAM30A was remarkably reduced and the level of miR-21-3p was dramatically elevated in CRC cells versus normal cells. (C) Regulation of FAM30A luciferase activity by miR-21-3p. miR-21-3p mimic suppressed the luciferase activity of FAM30A, while miR-21-3p inhibitor increased the luciferase activity of FAM30A WT. (D) The regulation of FAM30A expression in CRC cells by cell transfection and (E) its effect on the expression of miR-21-3p. Raised FAM30A inhibited miR-21-3p level, and the repression could be attenuated by miR-21-3p mimic. miR-21-3p mimic showed no significant effect on the expression of FAM30A ^ns^*P* > .05, ^***^P < .001, ^****^P < .0001.

FAM30A was predicted to bind with miR-21-3p via lncRNASNP v3. Therefore, both FAM30A and miR-21-3p were detected in CRC cell lines by PCR. It was shown that FAM30A level was remarkably reduced (Figure 3A, *P* < .0001) and miR-21-3p was significantly increased (Figure 3B, *P* < .0001) in CRC cells compared to normal cells.

Subsequently, LoVo and SW837 cell lines were selected for further research of the link between FAM30A and miR-21-3p. It was discovered that the luciferase activity of FAM30A WT was dramatically suppressed by the overexpression of miR-21-3p in LoVo and SW837 cells, whereas the inhibitor of miR-21-3p enhanced the luciferase activity of FAM30A WT remarkably. There was no significant change in FAM30A MT (Figure 3C).

In addition, overexpression of FAM30A could diminish the level of miR-21-3p, and the suppression would be reversed by miR-21-3p mimic (Figure 3D). However, miR-21-3p mimic showed no significant effect on FAM30A level, demonstrating that miR-21-3p was downstream of FAM30A and was negatively regulated by FAM30A (Figure 3E).

### Effect of FAM30A and miR-21-3p on Colorectal Cancer Cells

**Figure 4.**
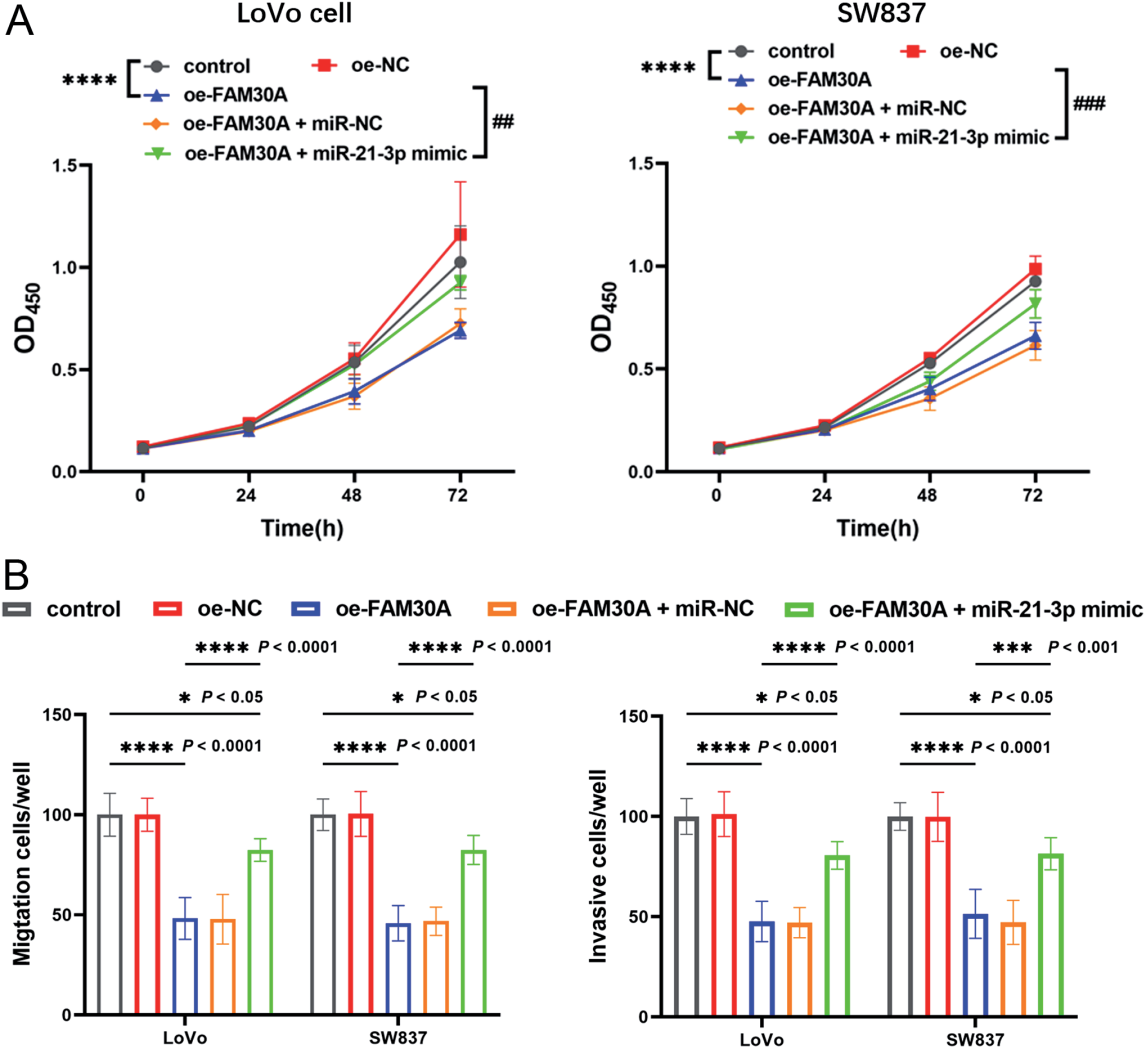
Influence of FAM30A and miR-21-3p on colorectal cancer (CRC) (A) cell proliferation and (B) metastasis. Increased FAM30A inhibits CRC cell proliferation, and this inhibition is attenuated by miR-21-3p mimic. Raised FAM30A also inhibited the migration and invasion of CRC cells, and this inhibition was diminished by miR-21-3p mimic ^***^P < .001, ^****^P < .0001 relative to control, ^##^*P* < .01, ^###^*P* < .001 relative to oe-FAM30A.

In comparison with the control, overexpression of FAM30A dramatically inhibited the proliferation of LoVo and SW837 cells, while the inhibition was attenuated by overexpressing miR-21-3p (Figure 4A). Additionally, the migration and invasion of LoVo and SW837 cells were also inhibited by a high level of FAM30A, which was also reversed by miR-21-3p mimic (Figure 4B).

## Discussion

This study revealed the significant downregulation of FAM30A in CRC, consistent with the data from GEO database and public starBase databases. Decreasing FAM30A predicted the malignant development and adverse prognosis of CRC patients. FAM30A also served as a tumor suppressor inhibiting cell growth and metastasis via negatively modulating miR-21-3p.

Colorectal cancer is the most prevalent gastrointestinal cancer with a high incidence and mortality rate, and it is prone to recurrence and metastasis, making the prognosis worse. Hence, learning about the pathogenesis of CRC and exploiting new therapeutic targets has become a focal point in the field of oncology research. As is well known, lncRNAs have also been reported to predict the outcomes of patients with various cancers, especially digeative cancer.^24^ The lncRNAs play an essential role in the progression of CRC, which could interfere with CRC cell proliferation, invasion, tumor growth, and metastasis.^25^ For example, lncRNA IGFL2-AS1 was identified as a tumor promoter in CRC that promoted malignant growth of HCT116 cells via modulating the miR-433-3p/PAK4 axis.^26^ FAM30A has received widespread attention in other tumors. For instance, FAM30A acts as an enabler in acute myeloid leukemia and laryngeal squamous cell carcinoma.^13,15^ Besides, FAM30A has been identified as a positive prognostic factor in gastric cancer, laryngeal squamous cell carcinoma, and lung adenocarcinoma.^12,13^ Meanwhile, our research also supports the critical prognostic value of FAM30A as an independent prognostic factor, which indicates that FAM30A could be a prognostic marker for CRC. LncRNA is an integral part of the ceRNA network and could modulate protein coding and translation in cancer by binding miRNAs.^19,20^ miR-21-3p, has been identified to be expressed in multiple cancers and correlated with the extent of tumor malignancy, considered to be a neoplastic focus of malignant tumors. In ovarian cancer, miR-21-3p regulates PEG3 expression and in turn regulates ovarian cancer progression.^27^ In gastric cancer, lncRNA GAS8-AS1 could restrain cancer cell proliferation by down-regulating miR-21-3p.^28^ In CRC, miR-21-3p promotes cell movement through modulating EMT.^21^ Low level of miR-21-3p could target RBPMS in HCT116 cells via the Smad4/ERK signaling pathway, which suppressed CRC cell proliferation, invasion, and migration, as well as induced cell apoptosis.^22^ Our research has discovered that miR-21-3p was a downstream factor of FAM30A and mediates the regulation of CRC progression by FAM30A. A high level of FAM30A could suppress CRC cell proliferation and metastasis, and the tendency could be mitigated by increased miR-21-3p.

Our research uncovered the vital role of FAM30A in the prognostic forecasting of CRC and exposed the potential molecular mechanism of FAM30A, which suggests the crucial role of lncRNAs in the progression of tumor-related research. In addition, qRT-PCR is a comparatively sensitive approach to detecting the levels of FAM30A and miR-21-3p. The consistency of ncRNA levels in clinical specimens improves the efficiency and specificity of the analytical method.

Nevertheless, there remain several limitations. First, study subjects were enrolled from a single center, and the sample size was not large enough, which may limit the relevance of the clinical results. In addition, the source of lncRNAs and miRNAs could also contribute to its function, such as exosomes and other microparticles.^29,30^ Hence, in the future, greater focus should be devoted to multicenter data with larger sample sizes and functional differences in ncRNAs from different sources. Besides, more in vivo substantiation of the in vitro findings will be required. Currently, animal models are the most valid in vivo research method, especially for tumor progression studies.^31^ Thus, future research requires the incorporation of animal experiments.

In conclusion, downregulated FAM30A in CRC predicted poor prognosis and severe development of patients and served as a tumor suppressor via negatively modulating miR-21-3p.

## Figures and Tables

**Table 1.** The Association of FAM30A Expression with Patients’ Clinicopathological Features

Characteristic	n	FAM30A Expression		*P*
		Low	High	
Total	107	55	52	
Age (years)				.778
≤60	52	26	26	
>60	55	29	26	
Gender				.702
Male	68	34	34	
Female	39	21	18	
Tumor size (cm)				.069
≤5	48	20	28	
>5	59	35	24	
TNM stage				.022
I-II	73	32	41	
III	34	23	11	
Location				.386
Colon	54	30	24	
Rectum	53	25	28	
CEA (ng/mL)				.027
<5	46	18	28	
≥5	61	37	24	
Differentiation				.043
Well–moderate	70	31	39	
Poor	37	24	13	
